# Assessment of CPME as Sustainable Low VOC Alternative to Hexane: Optimization of Extraction Efficiency and Bioactive Compound Yield from Fenugreek Seed Oil Using Computational and Experimental Methods

**DOI:** 10.3390/foods13233899

**Published:** 2024-12-03

**Authors:** Ameni Ben Abdennebi, Emna Chaabani, Mariem Ben Jemaa, Majdi Hammami, Saber Khammassi, Salma Nait Mohamed, Wissem Aidi Wannes, Ibtissem Hamrouni Sellami, Anne-Sylvie Fabiano Tixier, Iness Bettaieb Rebey

**Affiliations:** 1 Laboratory of Aromatic and Medicinal Plants, Borj Cedria Biotechnology Center, BP 901, Hammam-Lif 2050, Tunisia; benabdennebiameni@yahoo.fr (A.B.A.); emna.chaabani@cbbc.rnrt.tn (E.C.); mariembenjemaa@yahoo.fr (M.B.J.); hammamimajdi@hotmail.com (M.H.); saber.khammassi@cbbc.rnrt.tn (S.K.); aidiwanneswissem18@gmail.com (W.A.W.); hamrouni.ibtissem@gmail.com (I.H.S.); 2 Laboratory of Olive Biotechnology, Borj Cedria Biotechnology Center, BP 901, Hammam-Lif 2050, Tunisia; salma.nait@cbbc.rnrt.tn; 3 Avignon Université, INRAE, UMR SQPOV, F-84000 Avignon, France

**Keywords:** fenugreek, green solvent, oils, fatty acids, sterols, tocopherols, bioactivities

## Abstract

This study investigates the performance of cyclopentyl methyl ether (CPME) in the extraction of fenugreek seed oil, aiming to replace the conventionally used hexane. The efficiency of this alternative solvent was evaluated first through in silico methods (based on Hansen Solubility Parameters (HSPs) and Conductor-like Screening Model for Real Solvent (COSMO-RS) simulations), followed by experimental studies. Solubility computational predictions analysis revealed that CPME exhibits superior solvation power compared to hexane. Experimentally, CPME demonstrated a significantly higher oil yield (7.23%) compared to hexane (4.25%) and a better retention of beneficial unsaturated fatty acids than hexane. Additionally, the physicochemical properties of oils extracted with CPME showed enhanced oxidative stability, sterol, tocopherol, and phenolic contents, leading to superior antioxidant and antibacterial activities. Importantly, CPME’s low volatile organic compound (VOC) emissions further establish it as a more sustainable and environmentally friendly alternative to hexane, aligning with contemporary goals of reducing harmful emissions in extraction processes. Thus, this paper highlights the functional advantages of CPME, focusing on its efficiency, selectivity, and enhanced retention of bioactive compounds, positioning it as a superior extraction solvent for fenugreek seed oil compared to hexane.

## 1. Introduction

Oilseeds are essential raw materials for a wide range of industries, supporting applications in food production and in cosmetics, varnishes, adhesives, lubricants, soaps, synthetic resins, greases, paints, and waxes [1]. The extraction method employed significantly influences the quality of the final products and carries implications for environmental sustainability [2]. Traditionally, hexane has been the solvent of choice in oil extraction due to its effectiveness in dissolving a broad spectrum of lipophilic compounds. Its low boiling point makes it easy to remove the solvent after extraction, which is crucial for preserving sensitive compounds. However, concerns about hexane’s environmental impact and potential health risks have led to an interest in alternative solvents. Cravotto et al. [2] highlighted the importance of transitioning away from hexane, noting that while it offers immediate extraction benefits, its long-term consequences warrant further investigation into greener substitutes. Oilseed extraction holds importance beyond industrial applications. It plays a crucial role in the food industry, enhancing the nutritional and sensory qualities of various food products. As consumers increasingly prioritize health and sustainability, the demand for oils extracted using environmentally friendly methods is on the rise. Consequently, the use of green solvents in oil extraction has gained prominence due to growing concerns over the environmental and health impacts associated with conventional organic solvents [3]. Recognized for their eco-friendly characteristics, green solvents utilize renewable feedstocks and minimize emissions of organic solvents into the environment [4]. They also contribute to reduced extraction time, solvent, and energy consumption without compromising the recovery and quality of the oil extracts [3,5]. Among these, cyclopentyl methyl ether (CPME) stands out for its eco-friendly profile and versatile physicochemical properties, including a moderate polarity and boiling point, which enhance its efficacy in dissolving lipophilic compounds efficiently [4]. Furthermore, CPME reduces extraction time and solvent use while maintaining the quality and yield of oil extracts, making it a viable candidate for sustainable extraction processes. Fenugreek (*Trigonella foenum graecum* L.) seeds contain 5–7% oil, mainly composed of linoleic acid, linolenic acid, and oleic acid [6]. Fenugreek seed oil is used in flavoring many canned foods and syrups and as an ingredient in some perfumes [7]. The use of conventional solvents for oil extraction from fenugreek seed oil has been successfully evaluated [6,8,9,10,11]. Additionally, the extraction of fenugreek seed oil using supercritical CO_2_ (SC-CO_2_) has been extensively studied, demonstrating its effectiveness and optimization under various conditions [12,13,14]. Studies comparing CPME with conventional solvents, including hexane, ethanol, and chloroform, indicate that CPME can achieve similar or even superior extraction efficiencies while reducing overall solvent and energy consumption [13,14,15,16,17]. Furthermore, unlike supercritical CO₂, which requires high-pressure equipment, CPME is effective under milder conditions, making it more accessible for a range of applications [4]. Accordingly, this paper presents a comprehensive investigation into the use of CPME as an alternative solvent for fenugreek seed oil extraction, aiming to compare its yield, physicochemical properties, and bioactivities with those obtained through hexane. A computational approach was followed by experimental validation, positioning CPME as a promising sustainable extraction solvent with broader implications for food safety, quality, and consumer preferences in the evolving field of food production.

## 2. Materials and Methods

### 2.1. Reagents and Solvents

Solvents used were of analytical grade and were provided by VWR international (Darmstadt, Germany). CPME and standards used for chromatography analyses were purchased from Sigma-Aldrich Co, St. Louis (MO, USA). All other chemical reagents were obtained from Alfa Aesar Co. (Ward Hill, MA, USA) or Merck KGaA (Darmstadt, Germany).

### 2.2. Plant Material

*Trigonella foenum-graecum* seeds were harvested in June 2023 from Korba region in the northeastern part of Tunisia (latitude 36°34038.2200 (N); longitude 10°51029.6300 (E); altitude 637 m). Post-harvest, the seeds were stored in clean, dry bags to preserve their quality until extraction. For consistent and efficient extraction, the seeds were finely ground into a uniform powder using a blender.

### 2.3. Computational Methods

#### 2.3.1. Hansen Solubility Parameters (HSPs)

The HSPs were used to evaluate the ability of CPME to dissolve major hydrophobic components of fenugreek seed oil as a green substitute for hexane. This approach is based on the concept that the total cohesive energy density (δ_total_) is approximated by the sum of the energy densities required to overcome atomic dispersion forces (δ_D_), molecular polar forces arising from dipole moments (δ_P_), and hydrogen bonds (δ_H_).

This is expressed as

$$\delta_{total}^{2}=\delta_{D}^{2}+\delta_{p}^{2}+\delta_{H}^{2}$$

The relative energy difference (RED), calculated as the ratio between R_a_ (the distance between two materials based on their partial solubility parameters) and R_o_ (the radius of the solubility sphere), indicates the affinity between solute and solvent.

$$\mathrm{RED}=\frac{Ra}{R0}$$

A RED number less than 1.0 indicates high affinity, while numbers greater than 1.0 indicate low affinity. The HSPiP software, version 4.0 (Hansen-Solubility, Hørsholm, Denmark), using the Yamamoto Molecule Breaking (Y-MB) method, calculates HSP values and estimates physicochemical parameters like melting point and vapor pressure. This method provides reliable predictions for complex inter-group interactions and follows the “*like dissolves like*” rule, where a smaller R_a_ signifies a greater affinity between the solute and solvent.

#### 2.3.2. COSMO-RS Prediction

COSMO-RS is a powerful computational method that combines quantum chemical calculations (COSMO) and statistical thermodynamics (RS) to predict thermodynamic properties without requiring experimental data. Developed by Klamt, COSMO-RS has been applied since 2000, particularly in the extraction of natural products, to estimate solubilities and chemical potentials of molecules in various solvents.

The method involves immersing a solute molecule in a dielectric medium, constructing a surface around it, and optimizing the distribution of electrostatic charges using density functional theory (DFT) to find the molecule’s most stable state. This charge distribution, represented as the σ-surface and further simplified into a σ-profile, characterizes the molecular interactions within a solvent. COSMO-RS then uses these profiles to interpret solute–solvent affinities and calculate the chemical potentials of molecular surface segments (σ-potentials) through the COSMOthermX, version C30, release 17.02. By determining the relative solubility of compounds such as palmitic acid, oleic acid, and others, the model ranks solvents based on their ability to dissolve these compounds, providing crucial insights for solvent selection in substitution of toxic solvent with alternative and green solvents.

The calculation of relative solubility is derived from the following equation:

$$\log_{10}{(x}_{j})=\log_{10}\left[\frac{\exp\left({µ}_{j}^{pure}-{µ}_{j}^{solvent}-\Delta G_{j,fusion}\right)}{RT}\right]$$

$$\mu_{j}^{pure}:chemical\;potential\;of\;pure\;compound\;j\;(Joule/mol)$$

$$\mu_{j}^{solvent}:chemical\;potential\;of\;j\;at\;infinite\;dilution\;(Joule/mol)$$

ΔGj,fusion:free energy of fusion of j (Joule/mol)

$$x_{j}:solubility\;of\;j\;(g/g\;solvent).$$

The relative solubility is determined at infinite dilution. The logarithm of the best solubility is set to 0, and all other solvents are ranked relative to the best or reference solvent.

### 2.4. Solvent Extraction of Fenugreek Seed Oil

The initial oil content of fenugreek seeds was evaluated using a Soxhlet extractor with specific solvents (hexane and CPME). For this purpose, 25 g of seeds were placed in the extractor and subjected to extraction with 100 mL of solvent for a period of 6 h, these conditions were optimized to ensure maximum yield and efficiency. The resulting solvent–oil mixture was then transferred to a round flask, and the solvent was removed using a rotary evaporator (Rotavapor) at a temperature of 40 °C. Finally, the resulting oil extract was stored at 4 °C to prevent degradation of the compounds, for subsequent analysis. This method allows for a precise measurement of the oil content in the seeds, using appropriate solvents to effectively extract lipidic compounds. Yield refers to the mass of extract determined after evaporation of the solvent. It is expressed, however, as a percentage of the initial mass of the extracted plant. The extract percentages were calculated using the following formula:

Extraction yield (%)=M/M0×100

### 2.5. Determination of Physicochemical Properties

The physicochemical properties such as acid value, refractive index, peroxide value, and iodine value of fenugreek seed oils were measured based on the analytical methods described in Regulations EC 2568/91 (European Commission Regulation, EEC/2568/91, 2003).

### 2.6. Oxidative Stability

Oxidative stability of fenugreek seed oil was measured by Rancimat (Metrohm Rancimat; Metrohm, Riverview, FL, USA) based on the method of Tabee et al. [18]. Briefly, 2.5 g of fenugreek oil was heated to 110 °C with an air flow of 10 L/h, conducted in triplicate. Volatile compounds generated during oxidation were captured in a flask containing distilled water. The conductivity of the water was automatically measured to monitor oxidation progression. The oxidative stability was identified at the point where the oxidation rate showed a sharp increase, with results reported in hours.

### 2.7. Fatty Acid Composition Analysis

Fatty acid composition was analyzed by gas chromatography (GC) after derivatization to fatty acid methyl esters (FAMEs) with a 2 M methanolic solution of potassium hydroxide [17]. Then, FAMEs were analyzed by GC using HP 6890 gas chromatograph (Agilent, Palo Alto, CA, USA) equipped with RT-2560 capillary column (100 m length, 0.25 mm i.d., 0.20 μm film thickness). Nitrogen was used as carrier gas. The initial oven temperature was held at 170 °C for 2 min, increased at a rate of 3 °C/min ramp to 240 °C, and finally held there for 15 min. The injector and detector temperatures were 225 °C. Individual fatty acids were identified by comparing their retention times with a certified FAME mix.

### 2.8. Micronutrient Analysis

#### 2.8.1. Tocopherol and Sterol Determination

The tocopherol content of fenugreek seed oil was determined according to ISO 9936 standard [19]. Thus, 1 g of fenugreek oil was extracted with methanol and methanol/isopropanol solutions. After evaporation, the residue was dissolved in a methanol/isopropanol/hexane mixture and analyzed for tocopherol content using RP-HPLC with a UV–Vis detector. Tocopherols were separated on a C18 column with a gradient of four solvents (acetic acid in water, methanol, acetonitrile, and isopropanol). The flow rate was 1 mL/min, and the injection volume was 20 μL, with compound identification based on retention times compared to standards. In addition, the quantification of sterol was performed according to ISO 12228-1 standard [20]. Hence, seed oil was saponified with 1 M KOH in methanol for 18 h at room temperature. Water was added, and the unsaponifiable fraction was extracted six times using a mixture of n-hexane and methyl tert-butyl ether (1:1, *v*/*v*). The solvent was removed under nitrogen at ambient temperature. The dry residue was dissolved in 0.2 mL of pyridine and then silylated with 0.8 mL of Sylon BTZ. Sterol derivatives were separated using a Trace GC Ultra with a DB-35MS capillary column. A 1.0 μL sample was injected in splitless mode with a 5 min injection time. The column temperature was initially set at 100 °C for 5 min, raised to 250 °C at 25 °C/min (held for 1 min), then increased to 290 °C at 3 °C/min and held for 20 min. The detector temperature was maintained at 300 °C, and hydrogen served as the carrier gas at a 1.5 mL/min flow rate. Sterols were identified by comparing their retention times to those of commercial standards, with results expressed in mg/g of oil.

#### 2.8.2. Total Phenolic Content

Phenolic compounds were extracted from fenugreek oil using the method described by Parry et al. [19]. In brief, 0.5 g of oil was mixed with 2.5 mL of methanol, vortexed, and centrifuged at 5000 rpm for 5 min. This process was repeated twice, and the combined supernatant was lyophilized and stored at −20 °C. The total phenolic content was determined using the Folin–Ciocalteu reagent. An aliquot of the extract was mixed with distilled water, then reacted with Folin–Ciocalteu reagent for 3 min. After adding Na_2_CO_3_ solution and incubating in the dark for 2 h, the absorbance was measured at 760 nm. Results were expressed as milligrams of gallic acid equivalent per gram (mg GAE/g) of oil, using gallic acid as the standard.

### 2.9. In Vitro Antioxidant Activity

Three different antioxidant assays (total antioxidant capacity, DPPH assay, and reducing power) were employed to evaluate the antioxidant activity of methanol extracts obtained from fenugreek oil.

#### 2.9.1. Total Antioxidant Capacity (TAC)

The total antioxidant capacity of methanolic extracts was evaluated through an assay of the green phosphate/Mo^5+^ complex [21]. The absorbance was measured at 695 nm against a blank. The total antioxidant activity was expressed as mg GAE/g oil.

#### 2.9.2. DPPH Free Radical Scavenging Activity

The potential of methanolic extracts of oils to reduce the free DPPH radical (1,1-diphenyl-2-picrylhydrazyl) was expressed as IC_50_ (g/mL), the antiradical dose required to cause a 50% inhibition. For that, each methanolic extract of seed oils was mixed with a methanolic solution of DPPH [22]. The mixture was vigorously mixed and left in darkness for 90 min. The absorbance was measured at 517 nm against pure methanol using a UV–Vis spectrophotometer.

A total of 0.5 g of oil was subjected to extraction using 2.5 mL of methanol. The mixture was agitated and then centrifuged at 14,000× *g* to separate the components. The supernatant was carefully collected, and this extraction procedure was performed two additional times to ensure complete recovery of polyphenols. The combined extracts were then processed by dissolving 125 µL of the sample in 500 µL of distilled water, followed by the addition of 125 µL of Folin–Ciocalteu reagent, mixing vigorously to ensure homogeneity. To this solution, 1.25 mL of 7% sodium carbonate was added, along with distilled water to reach the desired volume. The resulting mixture was incubated in the dark for 90 min. Finally, the absorbance of the solution was measured at 760 nm using a UV–Vis spectrophotometer, and the total polyphenol content was calculated as milligrams of gallic acid equivalents per gram of oil (mg GAE/g).

#### 2.9.3. Reducing Power Assay

The ferric reducing antioxidant power (FRAP) of oil extracts was determined following the method described by Benzie and Szeto [23]. In fact, three milliliters of FRAP reagent was mixed with the 10 µM of each polyphenolic solution, and the content was mixed vigorously. The absorbance was read at 593 nm at intervals of 30 s for 4 min. An aqueous solution of known Fe 2+ concentration in the range of 100–1000 µmol/L was used for calibration.

### 2.10. Antimicrobial Activity

The effect of using a green extraction method on the antibacterial efficiency of fenugreek oil was evaluated, according to Petropoulos et al. [24], with slight modifications, through the micro-dilution technique against different pathogenic bacteria, namely *Entrococcus faecalis* (ATCC 29212), *Staphylococcus aureus* (ATCC 25923), *Escherichia coli* (ATCC 8739), and *Salmonella thyphimirium* (ATCC 14028).

To determine their respective MIC (Minimum Inhibitory Concentration) and MBC (Minimum Bactericidal Concentration), serial dilutions of hexane and CPME fenugreek oil extracts were prepared in aqueous solution of DMSO (10% *v*/*v*) and placed in wells inoculated with 10 µL of standardized bacterial suspension (10⁶ CFU/mL) and Muller–Hinton broth. The 96-well plate was then incubated overnight at 37 °C. The highest dilution at which no growth was observed was recorded as the MIC. For the MBC test, aliquots (200 µL) of the culture medium from wells without growth were spread on agar and incubated again overnight at 37 °C. The highest dilution with no survivors was recorded as the MBC.

### 2.11. Statistical Analysis

The results from the analytical methods are presented as the mean ± standard deviation (SD). To identify significant differences among the means of experiments conducted in triplicate, a Duncan test was applied with a significance level of *p* < 0.05.

## 3. Results and Discussion

### 3.1. Computational Methods

The solubility of the primary components of fenugreek seed oil in hexane and CPME was assessed using two computational approaches: HSP and COSMO-RS simulations. Table 1 provides a comparative overview of the chemical structures and properties of the selected solvents, including their energy efficiency, toxicity, resource usage, and carbon dioxide footprint, with hexane serving as the reference solvent. Despite differences in molecular weight, Table 1 indicates that CPME and hexane have similar refractive indices, boiling points, enthalpies of vaporization, and surface tensions, suggesting comparable energy requirements for solvent recovery. Additionally, CPME exhibits a relatively high log *p* value, reflecting its lipophilic properties.

### 3.2. HSPs

The ability of hexane and CPME to solubilize the main fatty acids of the fenugreek seed oil is represented by the RED (Table 2). The RED value is calculated as the ratio of the HSP distance between the solvent and a reference value to the radius that defines the effective solubility. A solvent with a RED value of ≤1 is considered to have good miscibility with the target solute, while a solvent with a RED value >1 is regarded as having poor miscibility. The results indicate that CPME, with a RED value < 1, exhibits a higher solvation power compared to hexane, making it theoretically more effective for isolating these metabolites from fenugreek seeds. These findings align with prior research that has highlighted the potential of CPME as an eco-friendly alternative to hexane for extracting edible oils from various sources, including *P. lentiscus* berries [15], sesame seeds [17], and carrots [25].

### 3.3. COSMO-RS Prediction

The solubility of the major fatty acids of fenugreek oil in hexane and CPME was predicted using the COSMO-RS tool. Table 2 presents the relative solubility (log_10(X_RS)_), which establishes the logarithm of the highest solubility as 0. CPME exhibited a significantly higher theoretical solubility for all solutes, with the relative solubility of the five free fatty acids considered equal to 0.

CPME exhibited a significantly higher theoretical solubility for all solutes, with the relative solubility of the five free fatty acids considered equal to 0. Based on the principle of “*like dissolves like*”, CPME appears to offer a promising alternative solvent to hexane for extracting all studied compounds. These findings are in accordance with previous works reporting that CPME could theoretically be a promising alternative to hexane in the extraction of oil from different sources [15,17,26,27]. This initial in silico approach marks a crucial first step in assessing CPME’s potential as a replacement for hexane in fenugreek oil extraction.

### 3.4. The Effect of CPME on the Extraction of Fenugreek Oil

#### 3.4.1. Oil Yield

The fenugreek oil yield obtained using CPME and hexane is illustrated in Table 3. CPME demonstrated a significantly higher extraction efficiency, yielding 7.23% compared to hexane’s 4.25%. This performance could be attributed to the solubility properties and molecular interactions of CPME with the lipophilic components in fenugreek seeds. Our findings are consistent with previous studies. Thus, Akbari et al. [11] conducted Soxhlet extraction using hexane as a solvent, yielding 5.55%. Similarly, Munshi et al. [28] reported extraction yields of 4.37%, 3.65%, 3.97%, and 3.65% using hexane, petroleum ether, acetone, and ethanol, respectively. Notably, Ren et al. [29] obtained the highest oil yield (8.95%) by employing supercritical CO_2_ fluids for fenugreek oil extraction. While hexane is commonly utilized as a solvent in vegetable oil extraction owing to its efficiency and cost-effectiveness [4], its associated risks of explosion, toxicity, and environmental impact present notable constraints, especially concerning organic food products [30]. Consequently, regulations have been implemented to restrict hexane residue levels in food (Directive 2009/32/EC). Remarkably, CPME has emerged as a highly promising and non-toxic alternative to hexane. Despite its relatively higher cost, CPME has demonstrated exceptional efficacy in various extraction processes, suggesting its strong potential to replace hexane [15,17,27]. This underscores CPME’s power as a safer and more environmentally sustainable solvent for oil extraction. Therefore, the adoption of CPME could mitigate the health and environmental risks associated with hexane, aligning with the increasing demand for greener and safer solvent extraction [31].

#### 3.4.2. Fatty Acid Composition

**Figure 1:**
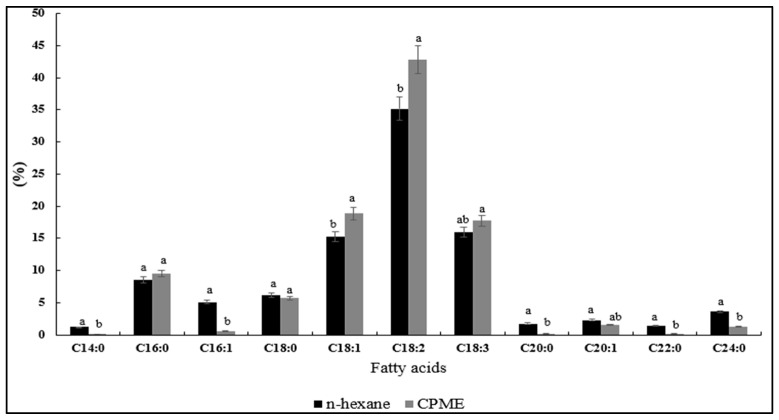
Fatty acid composition of fenugreek seed oils extracted with conventional and green solvent; a, b letters = significant differences at *p* (0.05).

Regarding the fatty acid composition, the analysis of fenugreek lipid fractions showed no significant selectivity between solvents (Figure 1). Twelve fatty acids were identified; among them, the predominant fatty acids include linoleic acid (C18:2) (35.18–43.78%), linolenic acid (C18:3) (15.93–17.71%), oleic acid (C18:1) (15.23–18.84%), and palmitic acid (C16:0) (8.55–9.59%), respectively, for hexane and CPME. Likewise, unsaturated fatty acids (UFAs) constitute 73.76% to 82.51% of the total fatty acids in fenugreek oil when extracted with hexane and CPME, while saturated fatty acids range from 16.06% to 22.74%. Thus, fenugreek oil can be classified as a polyunsaturated oil. As a matter of fact, the analysis of the polyunsaturated to saturated fatty acid (PUFA/SFA) ratios indicates that CPME is superior to hexane for extracting lipids from fenugreek seeds, offering greater efficiency and yielding more beneficial UFAs. Our findings are in line with the fenugreek fatty acid profiles reported by Gu et al. [12] and Akbari et al. [11]. Moreover, several studies have reported that CPME has a high solvent power and extraction efficiency for lipophilic natural products and food ingredients [14,15,17]. The higher solubility power of CPME compared to hexane can be attributed to its enhanced solute–solvent interactions, structural properties, and its effectiveness in extracting lipophilic substances. This highlights CPME’s potential as a more efficient and eco-friendly substitute for extracting high-quality oils.

### 3.5. Analysis of Fenugreek Oil Extracted by Alternative Solvent

#### 3.5.1. Physiochemical Properties

As shown in Table 3, the physicochemical properties of fenugreek seed oils did not exhibit significant differences between hexane and CPME extractions. The acid value (AV) is an important parameter in assessing the quality of oils, indicating the amount of free fatty acids present, which can affect the oil’s stability, taste, and shelf life [32]. In this study, the AV for fenugreek seed oil was determined to be 1.62 and 1.59 mg KOH/g oil, respectively, for hexane and CPME extracts. Generally, a lower AV indicates higher oil quality, characterized by reduced free fatty acid content [33]. Furthermore, the peroxide values (PV) were 0.71 mEq O_2_/kg for hexane and 0.92 mEq O_2_/kg for CPME.

These values indicate that both solvents are effective in limiting the oxidative deterioration of the oils during extraction. Additionally, the relatively low PV indicated the low degree of lipid oxidation in fenugreek oils, which suggests the presence of minor compounds with antioxidant activities [34]. In fact, the iodine value (IV) of CPME-extracted oil was considerably higher (149.03 g I2/100 g) compared to hexane-extracted oil (133.92 g I2/100 g). This indicates that CPME extracts a greater proportion of unsaturated fatty acids, which are advantageous for health. Likewise, both oils had similar refractive index (RI), suggesting that the fundamental properties of the oils remain consistent regardless of the solvent used.

Obviously, edible oils have a large amount of unsaturated fatty acids, so their iodine levels will be higher at higher unsaturation. Hence, a higher degree of unsaturation is related to increased lipid oxidation [35]. Furthermore, one of the main quality attributes of vegetable oils is their oxidative stability (OS), which is a very important parameter since it evaluates the oil’s resistance to oxidation under heat and light exposure [36]. Therefore, OS can be considered a valuable tool for oil ranking and a primary index for the identification of fraud in edible oils. In this study, the OS of fenugreek seed oil was analyzed by Rancimat. This method measures the oil’s capacity to resist peroxidation, indicated by the induction period. CPME-extracted oil showed better oxidative stability (3.95 h) compared to hexane-extracted oil (2.73 h). This indicates that CPME extracts a higher yield of oil while also producing oil with improved resistance to oxidative degradation. This is particularly important for prolonging the oil’s stability.

#### 3.5.2. Micronutrient Analysis

The properties of vegetable oils are closely linked to their sterol and tocopherol contents, which play a vital role in determining their antioxidant activity and shelf life. The sterol content of fenugreek seed oils extracted with hexane and CPME is provided in Table 4. The CPME extracts displayed higher concentrations of all measured sterols compared to hexane extracts. Specifically, cholesterol levels were 5.17 mg/kg in CPME extracts compared to 3.3 mg/kg in hexane extracts. Campesterol was 33.10 mg/kg in CPME extracts, while hexane extracts contained 22.76 mg/kg. Stigmasterol levels were 12.38 mg/kg in CPME extracts versus 8.09 mg/kg in hexane extracts. Lastly, β-sitosterol was present at 72.10 mg/kg in CPME extracts, compared to 53.26 mg/kg in hexane extracts.

The total sterol content was significantly higher in CPME extracts (122.75 mg/kg) compared to hexane extracts (87.41 mg/kg). Similarly, all the extracts exhibited similar total and individual tocopherol contents (Table 4). The results showed that α-tocopherol is predominant, with 643.94 mg/kg. β, γ, and δ-tocopherol together make up only about 25 mg/kg. The total tocopherol content was significantly higher in CPME extracts (735.94 mg/kg) compared to hexane extracts (669.4 mg/kg). The analysis of sterol and tocopherol contents in fenugreek seed oils demonstrates that CPME is a more effective solvent than hexane for extracting these bioactive compounds. This finding indicates improved extraction efficiency, better preservation of oil quality, and enhanced nutritional and health benefits. This result is consistent with the findings of Trad et al. [17], who reported that replacing hexane with bio-based solvents in the extraction of sesame oil does not alter the sterol and tocopherol composition.

#### 3.5.3. Phenolic Content

In this study, we quantified the total phenolic content (TPC) of fenugreek seed oils extracted using hexane and CPME solvents through spectrophotometric analysis. As shown in Table 5, the comparison between conventional and green extraction methods revealed that fenugreek seed oil extracted with the green solvent CPME exhibited a higher total phenolic content (TPC) of 14.83 mg GAE/g oil. In contrast, the oil extracted with hexane had a TPC of 12.50 mg GAE/g oil. These findings indicate that CPME demonstrates superior selectivity and yields oils with significantly higher TPC levels compared to hexane. This aligns with previous research by Trad et al. [16], who demonstrated CPME’s efficacy in extracting sesame seed oil rich in TPC (23.51 mg GAE/g) compared to hexane (8 mg GAE/g). Additionally, Akbari et al. [10] reported a TPC of 38.97 mg GAE/g oil for Malaysian fenugreek seed oil extracted using hexane.

#### 3.5.4. In Vitro Antioxidant Activity

As can be seen in Table 5, fenugreek seed oil extracted with CPME showed a significantly higher total antioxidant capacity (38.33 mg GAE/g) than that extracted with hexane solvent (35.75 mg GAE/g). Additionally, the extraction of fenugreek seed oil with CPME solvent showed the highest antiradical activity using DPPH (IC50 = 126.85 µg/mL) and ABTS (IC50 = 137.14 µg/mL) assays. However, the lowest antiradical activity was obtained with hexane solvent using DPPH (IC_50_ = 280 µg/mL) and ABTS (IC_50_ = 237.70 µg/mL) assays. Fenugreek seed oil extracted with CPME also showed higher reducing power activity (EC_50_ = 293.28 µg/mL) than hexane solvent (EC_50_ = 374.96 µg/mL). Indeed, the use of CPME enhanced the antioxidant potential of fenugreek seed oil compared to hexane extraction. This enhancement highlights CPME’s ability to extract beneficial components from fenugreek seeds, thereby improving its antioxidant properties compared to conventional solvents. Research by Trad et al. [17] similarly highlighted that CPME enhances the antiradical activity of sesame oil when compared to hexane. These findings suggest that CPME offers a promising alternative to n-hexane for extracting fenugreek seed oil with superior antioxidant properties. Additionally, Akbari et al. [10] reported strong antioxidant activity in fenugreek seed oil extracted with hexane. They demonstrated IC50 values of 172.60 µg/mL in the DPPH assay and 161.30 µg/mL in the ABTS assay. In general, our results suggested that CPME could be an excellent substitute for hexane to recover fenugreek seed oil with enhanced antioxidant activity.

#### 3.5.5. Antibacterial Activity

The capacity of both of the studied extracts to inhibit the growth of numerous pathogenic bacteria was compared in order to evaluate the effect of using a green solvent on the antibacterial efficiency of *T. foenum graecum* L. oil. Table 6 demonstrated the inhibitory and bactericidal effects of both hexane and CPME extracts of fenugreek seed oil expressed in terms of the Minimum Inhibitory Concentration (MIC) and Minimum Bactericidal Concentration (MBC) against a panel of Gram-positive and Gram-negative bacterial strains. The gathered results highlighted an interesting antibacterial efficiency of both of the studied extracts against all bacterial strains with different extents. Although the hexane extract of fenugreek oil presented similar bacterial growth inhibition capacities with an MIC of 50 mg/mL against all tested strains, it presented variability in its bactericidal effects. For instance, *E. feacalis* presented the highest sensitivity towards hexane extract with a bactericidal effect of 60 mg/mL, while *S. aureus* and *E. coli* were more resistant to hexane extract with a subsequent bactericidal effect at 70 mg/mL. This context underscores the potential of fenugreek oil as a natural therapeutic agent, including significant antibacterial activity. Indeed, numerous studies, which highlighted the interesting antibacterial efficiency of fenugreek oil against various microorganisms, explained the gathered results by pointing to the richness of fenugreek oil in unsaturated fatty acids (namely linoleic acid, linolenic acid, and oleic acid) and bioactive compounds (flavonoids, sterols, and tocopherols) [26,37,38,39]. The diverse modes of action exhibited by the distinct chemical ingredients of fenugreek oil are probably responsible for its observed antibacterial activity. For example, Wei et al. [40] reported that unsaturated fatty acids, by blocking bacterial fatty acid production, show strong antibacterial and antibiofilm effects, particularly against Gram-positive infections. According to Arjmand and Haeri [41], sterols can damage bacterial cell membranes and hinder enzymes essential for DNA replication and energy production. This disruption can lead to stunted growth and cellular death. Meanwhile, tocopherol can cause growth suppression and cell death by breaking down bacterial cell membranes and interfering with vital cellular functions [42].

Interestingly, the type of solvent used for extracting fenugreek oil did not influence the capacity of both extracts to inhibit the growth of each tested bacteria. Indeed, hexane and CPME extracts of fenugreek oil were able to inhibit the growth of the tested microorganisms with an MIC limited to 50 mg/mL. However, the bactericidal effect of the studied extract of fenugreek oil was positively influenced using the green extraction method. The gathered results showed that CPME extract presented higher bactericidal activity compared to hexane extract (except for *E. faecalis*). For instance, for the same bacterial strain (*S. aureus*), using CPME for fenugreek oil extraction decreased its MBC from 70 mg/mL (hexane extraction) to 50 mg/mL. Observed antibacterial activity enhancement could be explained by the fact that CPME fenugreek oil extract displayed higher concentrations of all measured sterols and tocopherol compared to the hexane extracts. Poudel et al. [42] explained that tocopherols and sterols exhibit antimicrobial activity that is concentration-dependent. Higher concentrations of these molecules result in greater bactericidal efficiency.

## 4. Conclusions

In summary, the extraction of fenugreek seed oil using CPME offers an alternative to traditional solvents like hexane, with a focus on reduced VOC emissions. However, it is important to note that CPME’s environmental impact, in terms of its carbon footprint and toxicity index, is not unequivocally lower than that of hexane. While CPME’s higher boiling point results in lower VOC emissions, it also requires more energy for Soxhlet extraction and solvent recovery, which could counterbalance its environmental benefits. Despite these considerations, CPME remains a valuable alternative due to its ability to extract bioactive compounds, such as polyunsaturated fatty acids and phytosterols, which are known for their antibacterial and antioxidant properties. These findings contribute to the advancement of green chemistry in food extraction and underscore the need for a nuanced assessment of eco-friendly solvents, taking into account both their operational efficiency and environmental trade-offs.

## Figures and Tables

**Table 1:** Solvent properties, energy efficiency, and toxicity of studied solvents.

	Hexane	CPME
Chemical structure	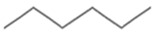	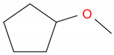
Solvent properties		
Mw (g.mol^−1^)	86.18	100.2
Density (g.mL^−1^)	0.66	0.86
Log P	3.48	1.7
R I	1.38	1.41
Flash point (°C)	−17	10
Melting point	−119.8	−122.2
Energy efficiency		
B.p (°C)	68.5	105.3
δHvap (KJ/mol)	28.9	33
Cp, liquid (KJ/mol*K)	0.195	0.2
Toxicity		
CMR *	2	5
Itox	No	4
Resource	Petroleum	Chemical synthesis
CO_2_ Footprint (kg.kg^−1^)	1.9	2

R I: refractive index; B.p: boiling point; Mw: molecular weight; * CMR classification: carcinogenic, mutagenic, and/or toxic to reproduction. Itox: toxicity index.

**Table 2:** Hansen solubility parameters (δ_d_, δ_p,_ and δ_h_ in Mpa^1/2^), RED values for HSPs, and COSMO-RS predicted solubilities for some target solutes found in fenugreek crude oils in hexane and CPME.

		Hexane	CPME
HSPs	δ_D_	13.9	16.7
	δ_P_	0.1	4.3
	δ_H_	0.1	4.3
Hansen (RED)	C16:0	2.39	0.69
	C18:0	2.15	0.67
	C18:1	2.32	0.62
	C18:2	2.58	0.72
	C18:3	2.59	0.62
COSMO-RS prediction (Log_10_ (X_solub))	C16:0	−1.27	0
	C18:0	−1.31	0
	C18:1	−1.34	0
	C18:2	−1.76	0
	C18:3	−1.80	0

**Table 3:** Oil yield and quality parameters of fenugreek seed oils extracted with conventional and green solvents.

	Hexane	CPME
Extraction yield (g/100 g DM)	4.25 ± 0.34 ^b^	7.23 ± 0.05 ^a^
AV (mg KOH/g oil)	1.62 ± 0.06 ^a^	1.59 ± 0.04 ^a^
PV (mEq O_2_/kg)	0.71 ± 0.01 ^a^	0.92 ± 0.00 ^a^
IV (g of I2/100 g)	133.92 ± 0.20 ^a^	149.03 ± 0.12 ^ab^
RI (20° C)	1.30 ± 0.03 ^a^	1.36 ± 0.02 ^a^
Oxidative stability (h)	2.73 ± 0.01 ^b^	3.95 ± 0.01 ^a^

The data marked with different superscript letters indicate significance at *p* < 0.05 (Duncan’s test).

**Table 4:** Sterol and tocopherol contents in fenugreek seed oils obtained by bio-based solvent compared to the reference.

	Hexane	CPME
	Sterol Contents (mg/kg oil)	
Cholesterol	3.3 ± 0.01 ^b^	5.17 ± 0.06 ^a^
Campesterol	22.76 ± 1.02 ^b^	33.10 ± 1.29 ^a^
Stigmasterol	8.09 ± 0.96 ^b^	12.38 ± 0.02 ^a^
β-Sitosterol	53.26 ± 1.23 ^b^	72.10 ± 1.04 ^a^
Total	87.41 ± 2.70 ^b^	122.75 ± 3.29 ^a^
	Tocopherol Contents (mg/kg of oil)	
α-Tocopherol	643.94 ± 0.13 ^ab^	703.01 ± 1.26 ^a^
β-Tocopherol	18.22 ± 3.11 ^ab^	22.76 ± 1.45 ^a^
γ-Tocopherol	5.30 ± 0.23 ^b^	7.14 ± 0.04 ^a^
δ-Tocopherol	1.94 ± 0.02 ^b^	3.03 ± 0.09 ^a^
Total	669.4 ± 0.56 ^b^	735.94 ± 0.23 ^a^

a, b letters = significant differences at *p* (0.05).

**Table 5:** Total phenolic (mg GAE/g) and antioxidant properties of fenugreek oil obtained by hexane and the bio-based solvent (CPME).

	TPC (mg GAE/g)	TAC (mg GAE/g)	DPPH (IC50 µg/mL)	Reducing Power (EC50 µg/mL)
Hexane	12.03 ± 1.02 ^b^	35.75 ± 0.63 ^a^	280 ± 0.92 ^b^	374.96 ± 2.03 ^b^
CPME	15.80 ± 2.71 ^a^	38.33 ± 0.04 ^a^	126.85 ± 1.83 ^a^	293.28 ± 1.93 ^a^

TPC: total phenolic content; TAC: total antioxidant potential; GAE: gallic acid equivalent; IC50: half maximal inhibitory concentration; EC50: half maximal effective concentration; a, b letters = significant differences at *p* < 0.05. IC50 values represent the mean of three replicates (n = 3); The data marked with different superscript letters indicate significance at *p* < 0.05 (Duncan’s test).

**Table 6:** Antibacterial activity of fenugreek oil.

	ATCC	Strains	MIC (mg/mL)	MBC (mg/mL)
		Hexane		
Gram (+)	29,212	*Entrococcus feacalis*	50	60
Gram (+)	25,923	*Staphylococcus aureus*	50	70
Gram (−)	3739	*Escherichia coli*	50	70
Gram (−)	14,028	*Salmonella thyphimirium*	50	ND
		CPME		
Gram (+)	29,212	*Entrococcus feacalis*	50	60
Gram (+)	25,923	*Staphylococcus aureus*	50	50
Gram (−)	3739	*Escherichia coli*	50	60
Gram (−)	14,028	*Salmonella thyphimirium*	50	50

## Data Availability

The original contributions presented in this study are included in the article. Further inquiries can be directed to the corresponding author.
